# Autonomous Car Parking System through a Cooperative Vehicular Positioning Network

**DOI:** 10.3390/s17040848

**Published:** 2017-04-13

**Authors:** Alejandro Correa, Guillem Boquet, Antoni Morell, Jose Lopez Vicario

**Affiliations:** Telecommunications and Systems Engineering Department, Universitat Autònoma de Barcelona, Bellaterra 08193, Spain; guillem.boquet@uab.cat (G.B.); antoni.morell@uab.cat (A.M.); jose.vicario@uab.cat (J.L.V.)

**Keywords:** vehicular communications, autonomous car, smart parking, vehicular sensor networks

## Abstract

The increasing development of the automotive industry towards a fully autonomous car has motivated the design of new value-added services in Vehicular Sensor Networks (VSNs). Within the context of VSNs, the autonomous car, with an increasing number of on-board sensors, is a mobile node that exchanges sensed and state information within the VSN. Among all the value added services for VSNs, the design of new intelligent parking management architectures where the autonomous car will coexist with traditional cars is mandatory in order to profit from all the opportunities associated with the increasing intelligence of the new generation of cars. In this work, we design a new smart parking system on top of a VSN that takes into account the heterogeneity of cars and provides guidance to the best parking place for the autonomous car based on a collaborative approach that searches for the common good of all of them measured by the accessibility rate, which is the ratio of the free parking places accessible for an autonomous car. Then, we simulate a real parking lot and the results show that the performance of our system is close to the optimum considering different communication ranges and penetration rates for the autonomous car.

## 1. Introduction

In the past decade, the scientific community has introduced the concept of Vehicular Sensor Networks (VSNs) where the connected and autonomous cars are seen as nodes of an heterogeneous sensor network [1]. This has been motivated by the development of Intelligent Transportation Systems (ITS) where the connected and autonomous vehicles are the central elements. These vehicles will communicate with each other within a Vehicular Ad hoc Network (VANET) through Dedicated Short-Range Communications (DSRC) based on the IEEE 802.11p standard and using the 5.9 GHz band. Furthermore, these new types of vehicles will include multitude of sensing technologies (cameras, ultrasound sensors, laser radars, inertial sensors) that provide relevant information for the ITS, such as the state of the traffic, detection of collisions or detection of available parking places. Therefore, connected and autonomous cars are considered as key elements for sensing the environment not only for the ITS but also for the smart cities [2,3] thanks to the characteristic mobility of cars that increases the coverage area of the sensor network.

Although the connected car of future VSNs is still at development, nowadays commercial cars already include wireless technologies such as WiFi. Indeed, Gartner foresees that for 2020 one in five vehicles will have some form of wireless network connection [4]. From now on, more intelligence will be added to vehicles, starting from driving aided systems, to connected cars and finally to the fully autonomous cars [5]. In fact, Ford has announced the intention to deliver a fully autonomous vehicle for ride sharing by 2021 [6].

The development of the automotive industry towards the VSNs has motivated new emerging vehicular applications [7]. Examples of applications go from drastically reducing the number of accidents to minimizing the transport cost or reducing the traffic congestion, among others like improving the driving experience or reducing the environmental impact of cars. Vehicular applications can be classified into three groups depending on its main objective [8]: (*i*) safety applications if their objective is to reduce accidents; (ii) traffic management applications if their objective is to clear up traffic; and (iii) value-added services for the rest of applications with objectives such as providing mobility to more people.

Recent advances in sensing and communications made possible systems that provide accurate and real-time detection of vehicle park space availability [9,10]. Usually, the detection of the occupancy of a parking place is done using sensors such as ultrasound sensors [11], magnetometers [12] or optical sensors [13]. On the value-added services category, parking place management applications intend to solve the time and energy consuming problem of finding available parking spaces. These applications also have to deal with management issues such as the reservation of places, the payment systems, the access control to the parking lots among others. For example, in [13,14], a Wireless Sensor Network (WSN) is deployed in a parking lot in order to monitor the occupancy of the parking. A VANET based smart parking system is presented in [15], providing real-time navigation for the cars and monitoring the occupancy of the parking lot. A similar system is found in [16], where the authors also include a mechanism for reserving a specific parking place. There are other works focusing on the design of methods for discovering free parking places in the streets of a city using ultrasound transmitters [11] or the design of methods for selecting the parking place for traditional cars in order to minimize the distance walked by the user from the parking place to its destination [17]. However, far too little attention has been paid to the development of applications based on VSNs that take into account the coexistence between the autonomous cars and the traditional cars.

As in any other Wireless Sensor Network (WSN) involving mobile nodes, the position of the nodes of a VSN must be known at any time because the sensor measurements are meaningless if the information about the position where they were taken is not available. Furthermore, in the particular case of VSNs, the position of the vehicle is also necessary for the autonomous cars to safely drive and park by itself [18]. Usually, vehicles deduce their position from the combination of GPS measurements with inertial measurements. However, Line Of Sight (LOS) to at least four positioning satellites is needed to obtain an acceptable position estimation, which limits the availability and the accuracy of the technology in GPS denied environments such as urban canyons or indoor parking lots. To circumvent the problems of GPS denied environments, widely used approaches in the literature to substitute the GPS measurements are the Received Signal Strength (RSS), the Time of Arrival (ToA) and the Time Difference of Arrival (TDoA) both in anchor based solutions and in cooperative approaches [19]. In the past decade, many vehicular guidance systems have been developed. An example can be found in [20] where the authors employ an anchor based solution by deploying Roadside Units (RSUs) all over a parking lot allowing parked vehicles to communicate space availability with each other by joining a VANET. These approaches require Vehicle to Vehicle (V2V) and Vehicle to Infrastructure (V2I) communications to periodically exchange state information and accurately estimate the vehicle position with the received RF signals from RSUs. The positioning accuracy of these systems depends directly on the number and placement of RSUs, which considerably increases the monetary cost of the system. For this reason, authors developed cooperative systems where, instead of using RSUs, the surrounding cars are considered as anchor nodes. Examples of cooperative systems can be found in [21,22].

In the actual paradigm, traditional, connected and autonomous vehicles will have to coexist for a certain period of time as the market evolves. Accordingly, to open new market opportunities, traditional parking lots should offer in their systems parking applications for autonomous vehicles as an added value. Therefore, it is interesting to design new solutions that allow autonomous vehicle users to coexist with traditional cars and park in the same parking lot with the minimum added infrastructure.

Within this framework, in this work, we take profit of our accumulated experience in WSNs [23,24,25,26] to design a self-automated parking lot on top of a VSN with a single RSU placed at the entrance of the parking. Upon entering the parking lot, vehicles use V2I communication to exchange the parking map and the information about available places (detected employing an underlying sensor network) with the parking RSU. Then, the system communicates to the vehicle which is the best parking place, selected with the aim of improving the accessibility to the parking for the next autonomous cars. The vehicle is guided to the assigned parking place thanks to the cooperation of all the autonomous vehicles in the parking lot. Note that parked autonomous vehicles can be used as anchors to improve the accuracy in position estimation. This strategy allows to mitigate the need to deploy RSUs because parked vehicles know their exact position, have the necessary technology and may remain parked for long periods of time. The main contributions of our work follow:

- Design of a smart parking system on top of a VSN with minimum added infrastructure considering that autonomous cars will coexist with traditional cars.
- Definition of the accessibility rate as a measure of parking place availability for the autonomous car.
- Design of a searching algorithm to select the best parking place for an autonomous car in terms of the accessibility rate.
- A simulation testbed that compares the results of our designed searching algorithm with the optimum case.

This paper is organized as follows. In Section 2, we introduce the parking model and the problem statement. The new design method for searching the best parking place is described in Section 3, whereas the simulation results appear in Section 4. Finally, the conclusions of this work are presented in Section 5.

## 2. Parking Model

In this section, we describe the problem statement and the metric used to decide whether an autonomous car can park or not in a specific parking place.

### 2.1. Problem Statement

Let us define an arbitrary indoor parking with *N* parking places with known positions $s_{i}={\left[\begin{array}{cc} x_{i} & y_{i} \end{array}\right]}^{T}$ for i=1,…,N defining a set,

(1) $$S=\{s_{1},s_{2},\ldots ,s_{N}\},$$

where $x_{i}$ and $y_{i}$ are the respective Cartesian coordinates. Let us also define the roads that give access to the parking places in a discrete manner as a set R containing all the positions $r_{i}={\left[\begin{array}{cc} x_{i} & y_{i} \end{array}\right]}^{T}$ that belong to the road, that is,

(2) $$R=\{r_{1},r_{2},\ldots ,r_{M}\},$$

where *M* is the number of total points in the road. In addition, the only infrastructure added to the parking is a single RSU with known position defined as:

(3) $$\mathrm{RSU}={\left[\begin{array}{cc} x_{RSU} & y_{RSU} \end{array}\right]}^{T}.$$

**Figure 1:**
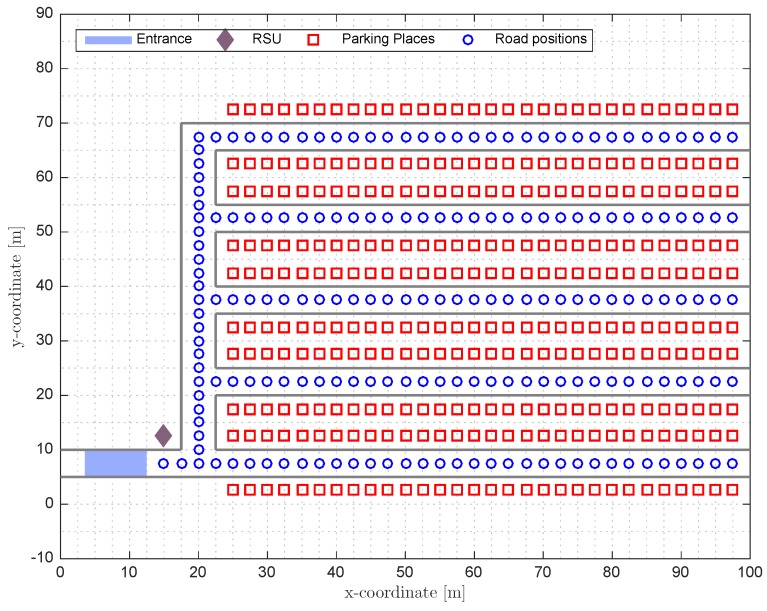
Simulated parking layout.

Figure 1 shows an example of a parking lot where the positions of the parking places are depicted as squares, the position of the RSU is depicted as a rhombus and the positions of the road points are depicted as circles.

In this work we consider two different kinds of cars. On the one hand the autonomous cars with V2X capabilities and driver-less, defining the set A, that is,

(4) $$A=\{a_{1},a_{2},\ldots ,a_{K}\},$$

where a∈S is the position where an autonomous car is parked. On the other hand, we define a set B of traditional cars without V2X capabilities, that is,

(5) $$B=\{b_{1},b_{2},\ldots ,b_{L}\},$$

where b∈S is the position where a traditional car is parked. We assume perfect knowledge of the sets A and B. Note that the autonomous cars will park in the parking places assigned by the system so the system knows at any time the position of the autonomous cars. In the case of traditional cars, we assume that the parking lot is able to detect which parking places are occupied. This can be done using one of the multiple technologies available in the literature, such us magnetic sensors [12] or optical sensors [13]. Note also that, currently, there are many parking lots that already include the technology to detect the occupancy of the parking space so no further infrastructure is needed. The sizes of sets A and B are defined by the occupancy rate $O_{rate}$ and penetration rate $P_{rate}$ of the parking. The $O_{rate}$ is the ratio between the number of occupied places and the total number of places, *N*, that is

(6) $$O_{rate}=\frac{L+K}{N},$$

where *K* is the size of set A and *L* is the set of B, respectively. Similarly, the $P_{rate}$ is the ratio between the number of autonomous cars and the total number of cars, that is,

(7) $$P_{rate}=\frac{K}{O_{rate}\cdot N}.$$

At a given time instant, cars of sets A and B will be distributed around the parking places so there will be $F=(1-O_{rate})N$ free parking places. Whenever a new car enters the parking, the set of free parking places available for this car will be different depending on the car type. If the new car belongs to set B, it is allowed to park in any of the free parking places. However, if the new car belongs to set A, it can only be parked on those free parking places where the car can access with enough positioning accuracy to be able to park without a driver. Note that the positioning accuracy is not only needed in the position of the parking place but all the way from the entrance to the parking place because the autonomous car is driving in a GPS denied environment; therefore, it has to be guided and rely on the system during all the path in order to avoid accidents.

In order to label a parking place as accessible for the autonomous car, we have to take into account all the positions in the path from the entrance to the parking place, so, for an arbitrary parking place $s_{j},$ we compute the path and create the set $P_{j}$ containing the indices of all the road points in R that are part of the path. Then, we verify that all the points in $P_{j}$ and the parking place $s_{j}$ fulfill the accuracy requirements of an autonomous car to park by itself. In that case, the parking place $s_{j}$ is labeled as accessible for the autonomous car.

Finally, the system will select the parking place for the autonomous car among all the free parking places that are accessible for the autonomous cars. Note that the selection of the parking place is not trivial because, once parked, the autonomous car will act as an anchor node for the incoming autonomous cars, and, therefore, it will increase the positioning accuracy of those incoming autonomous cars within its communication range. As a consequence, the number of free parking places accessible for the incoming autonomous cars may change after one new autonomous car is parked in the parking lot. For this reason, we introduce in this work the accessibility rate which measures the rate of free parking places that are accessible for incoming autonomous cars. We define the accessibility rate, $A_{rate}$, as the proportion of free parking places that are accessible for a new autonomous car, that is,

(8) $$A_{rate}=\frac{C}{F},$$

where *C* is the number of free accessible parking places for the autonomous car and *F* is the total number of free parking places.

### 2.2. Accuracy Requirements for Autonomous Parking

**Figure 2:**
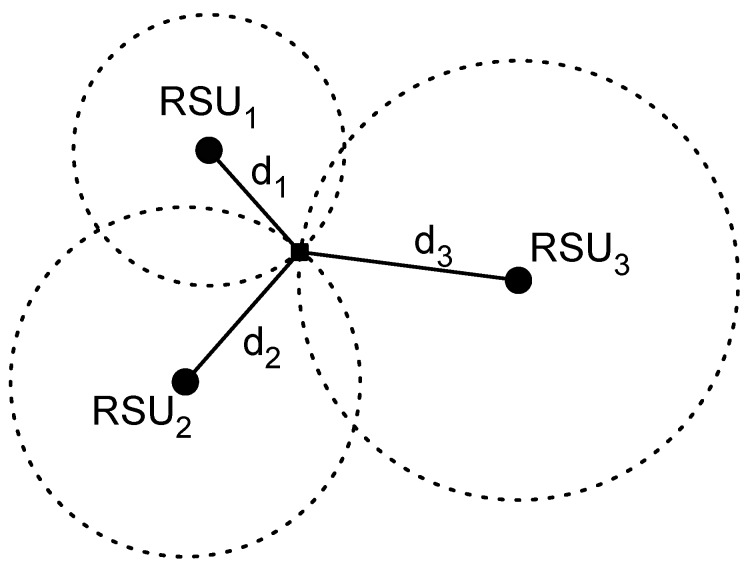
Lateration method concept.

The future autonomous car will compute its own position over time by combining multiple sources of position information such as GPS, inertial sensors, radars or vision based positioning systems. Although the position will be computed taking into account a large variety of sensors, it is expected that the burden of the position estimation will be for the GPS. Unfortunately, there are places where GPS cannot be used, like urban canyons or indoor scenarios; thus, new solutions must be considered. Commonly, this problem is circumvented by using the communications between the car and the RSU to first estimate the distance to the RSU and then, by combining the distance estimations to different RSUs, computing the position of the car using a lateration method. Lateration methods compute the position of a user as the intersection of different circles with the center as the anchor node position and radius as the estimated distance as depicted in Figure 2. In this case, the RSU is considered as an anchor node. For a two-dimensional position estimation, it is necessary to estimate the distance to at least three RSUs. Note that, if only two RSUs are employed, the circles will intersect at two different points, thus we cannot know in which of both points is the car without using additional information.

Recently, some authors have proposed to use the V2X capabilities of the autonomous cars to design cooperative positioning systems [21,27]. We follow this trend and, similarly in this work, surrounding autonomous cars will be used as anchor nodes in the lateration method.

Other sensors that will play an important role in the future positioning systems for the autonomous car are the inertial sensors. The inertial sensors measure physical quantities related to the motion of the car where the sensors are mounted. Typically, inertial sensors are grouped into an inertial measurement unit (IMU), which is formed by a three-axis accelerometer that measures the linear acceleration and a three-axis gyroscope that measures the angular velocity. The most general kind of inertial positioning system is the strapdown inertial navigation system. The idea beyond these systems is to estimate the position of the car by the double integration of the acceleration signal a(t). Thereby, the integration of the accelerometer signal results in the velocity, and, in turn, the integration of the velocity results in the position [28], that is,

(9) $$v\left(t\right)=v\left(0\right)+\int_{0}^{t}\left(a\left(t\right)-g\right)\;dt,$$

(10) $$m\left(t\right)=m\left(0\right)+\int_{0}^{t}v\left(t\right)\;dt,$$

where v is the velocity, g the gravity and m the position. The main disadvantage of inertial navigation systems is that the errors in the position estimations are successively accumulated by the integration procedure. Therefore, the position estimation accuracy decreases with time. Fortunately, it has been proved that the combination of inertial navigation systems with other positioning systems based on lateration such as the GPS or V2X based cooperative systems provide high accurate position estimations [29,30]. This fact has also been proved in other fields such as in indoor pedestrian navigation [31,32]. Furthermore, the combination of both systems relaxes the requirements on the number of RSUs available because the uncertainty generated by the multiple positions where the circles intersect is solved with the additional information given by the inertial navigation systems.

The design of a specific positioning system for the autonomous car is out of the scope of this work. Without loss of generality, in this paper, we assume that the autonomous car will compute its position employing the measurements from the on board inertial measurement unit and distance measurements to the different anchor nodes of the Vehicular Sensor Network, extracted employing periodic status exchange messages like Cooperative Awareness Messages (CAM) defined in the European Telecommunications Standards Institute (ETSI) G5 standard [33] or Basic Safety Messages (BSM) defined in the US Society of Automotive Engineers (SAE) standard [34]. In general, the positioning accuracy $a_{v}$ of a vehicle at a position, x, can be modeled as a function of the position itself and parameters related to both inertial and infrastructure-based positioning systems, p, that is,

(11) $$a_{v}=f\left(x,p\right).$$

Taking into account that the autonomous car parking and driving system requires a minimum accuracy $a_{v}^{th}$, it is then meaningful to search in the parameter space for all the values that attain $a_{v}^{th}$. Let us define this region as $P^{th}$.

It has been demonstrated in the indoor positioning field that, by combining inertial measurements with ranging measurements, it is possible to compute the position of a pedestrian with accuracies around 1 m [31,32,35,36,37]. It is then reasonable to expect that similar positioning systems will provide even better accuracies when mounted on vehicles because of the car motion model, which is simpler than the human motion, and also because cars can be provisioned with better inertial measurement units.

Without loss of generality, we consider this case as a realistic example to work with in the remaining of the paper. That is, we assume the system will attain positioning accuracy below 1 m when at least the distance measurements to two anchor nodes can be fused with the inertial data. In other words, $a_{v}=f\left(n_{a}\left(x\right)\right)$ where $n_{a}$ is the number of available anchor nodes and $P^{th}=\left\{n_{a}\left(x\right):n_{a}\left(x\right)\geq 2\right\}$. Notwithstanding, the ideas in this paper are also valid when we take into account other models for the positioning accuracy.

To compute the number of available anchor nodes in a specific position of the parking, $x={\left[\begin{array}{cc} x & y \end{array}\right]}^{T}$, we check for all the anchor nodes the following condition,

(12) $$||x-s_{j}{\left|\right|}^{2}<Radius,$$

where $s_{j}$ is the parking place where the anchor car is parked and Radius is its communication range.

For an arbitrary parking place $s_{j},$ we compute the path and create the set $P_{j}$ containing the indices of all the road points in R that are part of the path. Then, we can compute the set $E_{j}$ containing the number of anchors received at each point in the path, that is,

(13) $$E_{j}=\left\{n_{a}\left(x_{i}\right)|i\in P_{j}\right\}.$$

We decide if a given parking place *j* is accessible for the autonomous car using the following condition:

(14) $$\min E_{j}\geq 2.$$

## 3. Tree Based Searching Algorithm (TBSA)

In this section, we describe the designed TBSA for selecting the best parking place for an autonomous car. TBSA is applied following the next scenario: the human responsible for the autonomous car decides to park in a specific parking lot. At this moment, inside the parking lot, there will be a specific number of cars parked, both traditional cars and autonomous cars. Obviously, the new car can only be parked in free parking places, defined by the set F, which contains the indices of the free parking places. However, the autonomous car cannot be parked in all of the free parking places, as previously stated, and only a subset of the free parking places are available, G⊆F. Thus, a free parking place belongs to G if the following condition is fulfilled:

(15) $$j\in G\Leftrightarrow \min E_{j}\geq 2.$$

**Figure 3:**
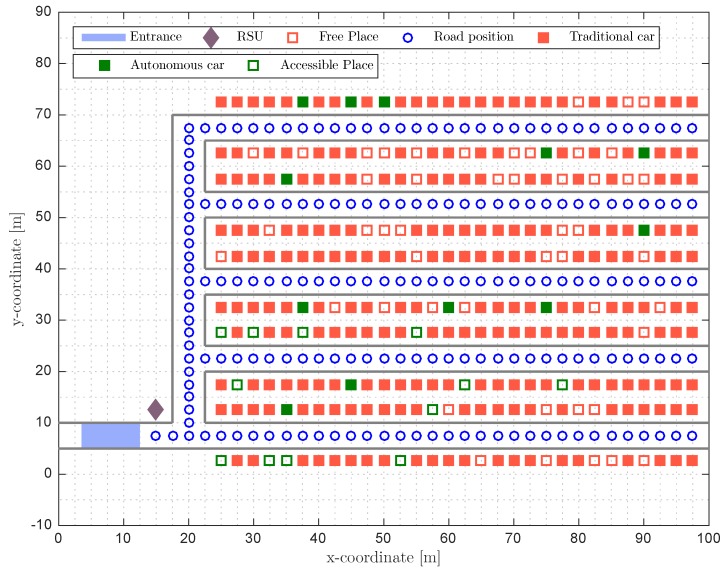
Parking state simulation with N=300, $O_{rate}=0.8$, $P_{rate}=0.05$ and $A_{rate}=0.2$.

Notice, however, that when an autonomous car parks in a specific place, the subset of free parking places available for the next autonomous car changes. Therefore, it is meaningful to design methods that grant the best possible parking conditions for the next autonomous cars. Several criteria can be used, but, in this work, we focus on the common good and we assume that the best parking place is the one that maximizes the accessibility rate (see Equation (8)) for the new cars. Note that, once parked, the new car will act as an anchor for other incoming autonomous cars and will increase the positioning accuracy of those cars inside its communications range. Figure 3 shows an example of this situation where the free parking places are marked as red unfilled squares and the free parking places accessible for the autonomous car are marked as green unfilled squares. Whenever a new autonomous car enters the parking, the system will select one of the free accessible parking places in order to maximize the $A_{rate}$ for future autonomous cars, that is,

(16) $$\max_{j\in G}\;A_{rate}\left(j\right)=\max_{j\in G}\;\frac{C(j)}{F-1},$$

where C(j) are the free accessible parking places once there is a new autonomous car parked in the parking place *j*. Note that the number of total free places, *F*, now is lower than in Equation (8) because now there is one more car parked in the parking. The computation of the optimum parking place involves the computation of the $A_{rate}$ for all the possible places where the autonomous car can be parked, that is, all the free accessible places, *C*, which can be a high complex process if the number of free accessible places is high. Note that for every free accessible parking place, we have to compute again the number of available anchor nodes at every position of the road, $r_{i}\in R$, and for every parking place, $s_{i}\in S$. Thus, if the complexity of one iteration of computing the available anchor nodes is α, the total complexity of the optimum algorithm will be Cα. For this reason, in this work, we design a suboptimal method with lower computational complexity.

**Figure 4:**
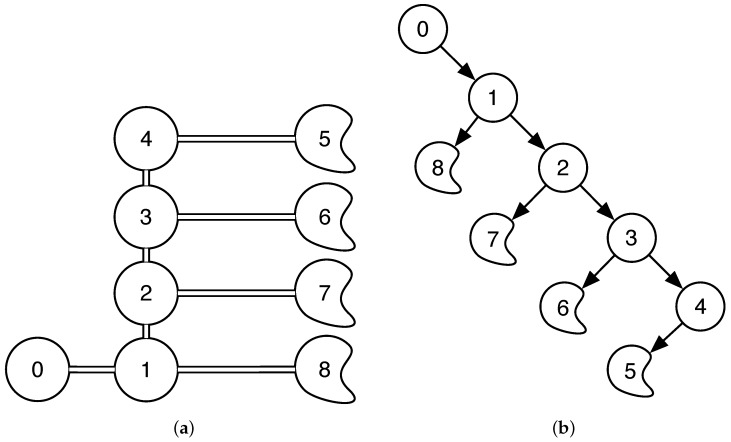
Simulated parking lot roads as trees: (**a**) undirected graph (**b**) directed out tree.

Let us express the roads of the parking as a tree where the nodes of the tree are the intersections and the ends of the roads as depicted in Figure 4a. In particular, we will define a directed out tree routed at node 0 [38] as depicted in Figure 4b, which corresponds to the entrance of the parking lot. Only the shortest path to a point in the parking is considered in the tree, that is, if one position of the parking can be accessed from different paths, only the shortest path will be included in the tree. For every road position of the map, we will store the arc of the tree that involves this position—for example, all the positions from the entrance to the intersection 1 will belong to the arc (0,1).

Remember that a parking place is only accessible for an autonomous car if the car can go from the entrance to the parking place with enough positioning accuracy. For example, for a parking place at node 7, all the way from the entrance to the node 7 must be covered with enough anchor nodes to have the desired positioning accuracy. This involves all the road positions associated to the arcs (0,1), (1,2) and (2,7). If any of these positions do not receive from enough anchor nodes, the parking place will be labeled as not accessible for the autonomous car.

**Figure 5:**
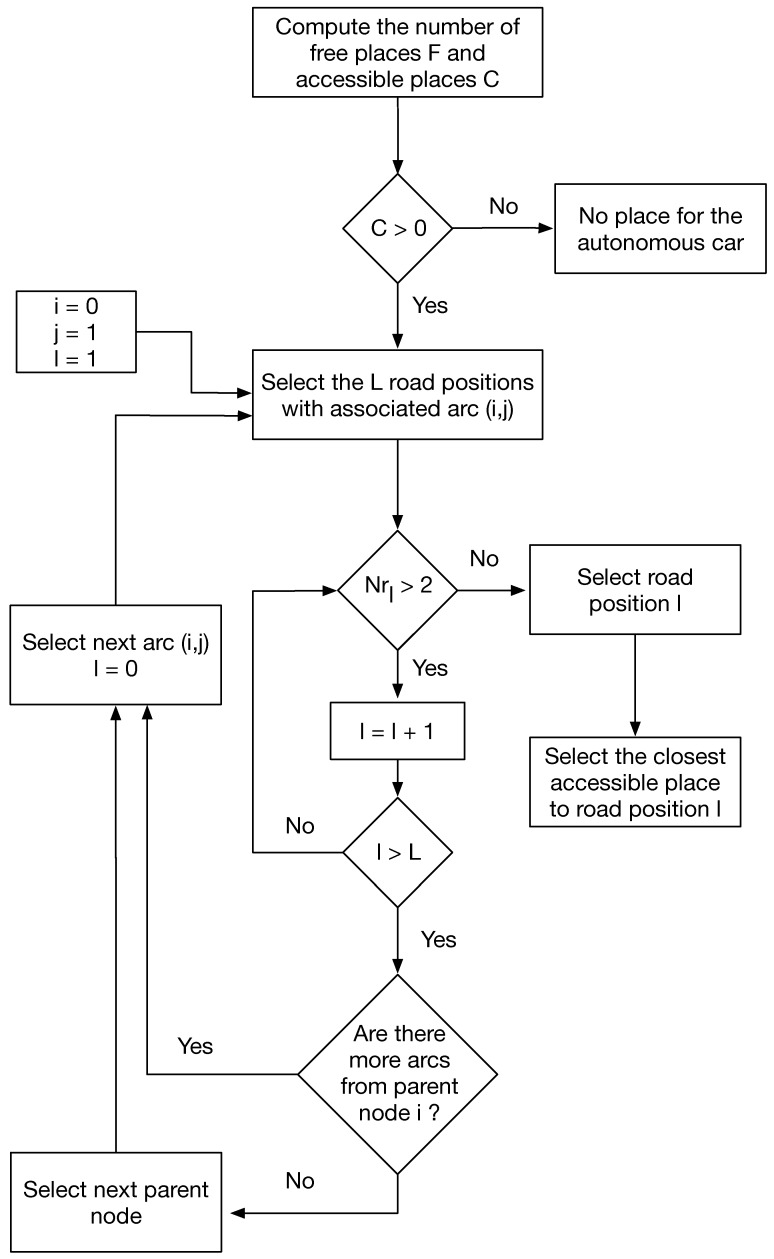
Tree Based Searching Algorithm (TBSA) block diagram.

Following this idea, we designed a method that covers all the directed out tree and selects the parking place for the autonomous car as the closest to the first point of the road that does not fulfill the anchor node condition. The block diagram of the designed method is depicted in Figure 5. We start from the entrance (node 0) and first look at all the points belonging to the first arc (0,1); if all of them receive from two or more anchor nodes, then we search in the following arc. In this case, it could be arc (1,2) or arc (1,8). By default, the method will choose the one involving lower numbers, but any of them could be selected. If, again, all the positions receive from more than two anchor nodes, we search for the following arc—in this case, arc (1,8). If all the road positions again fulfill the condition, we will search in the following arc—in this case, arc (2,3), as we already searched in all the arcs outgoing from node 1. Note that the algorithm will not select the arcs outgoing from node 2 until all the arcs from its parent node (node 1) are selected. The algorithm will follow this procedure until it detects one road position that receives from less than two anchor nodes. Once this position is found, we will select the closest free accessible parking place to the selected road position. The purpose of this algorithm is to create long directed paths inside the tree in order to give access to as many parking places as possible. As regards the computational complexity of the algorithm, note that, in the worst case, that is, when all the positions in the road fulfill the conditions, our algorithm will search the entire tree, which are the same number of positions that the optimum algorithm does at every iteration. Again, if we consider α, the computational complexity of one iteration, the computational complexity of the TBSA in the worst case is α, whereas the computational complexity of the optimum method is Cα. Therefore, the designed system, TBSA reduces the computational complexity by a factor *C* that depends on the number of free accessible places.

## 4. Simulation

In this section, we first describe the parking layout and then we present our simulation results. Our goal is to demonstrate the effectiveness of the method presented in this article comparing it with several other methods in terms of the accessibility rate.

In order to evaluate the performance of the proposed technique, the system is tested in one scenario based on the parking of the Engineering School at the *Universitat Autònoma de Barcelona* simulated using MATLAB in a Macbook Pro with a 2.2 GHz Intel i7 processor and 8 Gb of RAM. A layout of the simulated parking can be seen in Figure 1 and is composed of 300 places divided into five branches with 60 parking places each one. The only RSU available in the parking is placed at the entrance near the first intersection. This parking has only one entrance and one exit that are in the same place.The street belonging to the entrance and exit measures 100 m long while the other four streets oriented in the *x*-axis direction measure 77.5 m. On the other hand, the *y*-axis oriented street measures 60 m. All streets are 5 m in width. Finally, the distance between the centers of two consecutive places is 2.5 m like the distance between two consecutive road points. Note that the structure of many parking lots is similar to the one described here. There can be modifications in the number of branches of the parking, or the number of places, but, in general, any parking structure can be built by replicating the structure depicted here. Note also that, in the case of a parking with multiple entrances, we can create different trees for every entrance and apply the algorithm designed in Section 3 for the corresponding tree.

In order to test the performance of the designed TBSA method for the selection of the best parking place, we compare here four different methods: (i) the static method, that is, the stationary state of the parking before the autonomous car arrives and without introducing any new car; (ii) the random method, that is, a method that randomly selects the parking place for a new car; (iii) the TBSA described in Section 3; and (iv) the optimum method, that is, an exhaustive search algorithm that computes the $A_{rate}$ for all possible parking places and selects the optimum one.

To do so, we simulate a stationary state of the parking at a given time where the vehicles are randomly distributed around the parking places using a uniform distribution. As previously stated, we assume that the distribution of the vehicles around the parking lot is known by the RSU. Then, we compute the number of free places, free accessible places for the autonomous cars and the $A_{rate}$. From this situation, we compute the selected places for the random, TBSA and optimum methods and the accessibility rate is evaluated varying the occupancy rate, penetration rate and the ratio between the communication range and the maximum distance between two points of the parking lot. The last parameter of variation is selected because the effect of the communication range in the $A_{rate}$ depends on the size of the parking lot. In other words, the results obtained here can be extrapolated to larger parking lots and larger communication ranges. For the selection of the communications range, we adjusted the values following the results published in the literature based on measurement campaigns under the IEEE 802.11p standard [39,40]. Take into account that, although in general parking lots don’t usually have lots of walls that can block the signal, the communications will be done in a Non Line Of Sight (NLOS) environment. The walls are not the only object that can block a signal and produce NLOS conditions. In a parking lot, there will be columns and there will be some walls (for the stairs or elevators, for example), but, more importantly, there will be a lot of cars parked in the parking place and the cars will block the signal [41] and produce NLOS conditions. Note that one car can only partially block the signal, but if there is a series of cars parked side by side (which is the typical case for a parking lot), the blockage of the signal will be higher and the communications will be done in NLOS conditions. In particular, in the simulated scenario, we defined a communications range up to 60 m and the maximum distance of the parking lot is 105 m.

As we are dealing with a stochastic process while placing the cars, all results in this article are shown as the average of 10,000 iterations. In addition, because it is not in the scope of this article, it is assumed that the position estimations of vehicles are error free, which means that the autonomous car is always able to park while receiving from at least two anchors. It is also assumed that the communication range does not vary and is the same for all the nodes. Moreover, the communication range is referred to as Radius in the simulations for abbreviation reasons.

**Figure 6:**
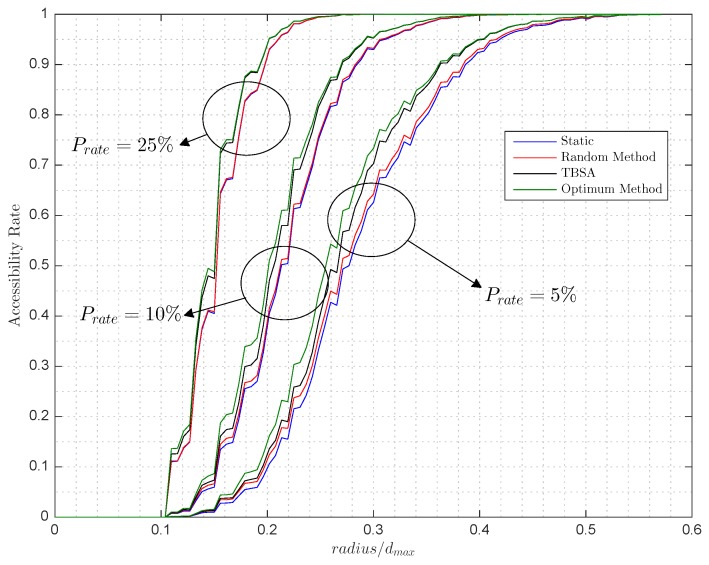
Simulated accessibility rate versus radius$/d_{max}$ with an occupancy rate of 80% for a penetration rate ($P_{rate}$) of (*i*) 5%, (ii) 10% and (iii) 25%.

**Figure 7:**
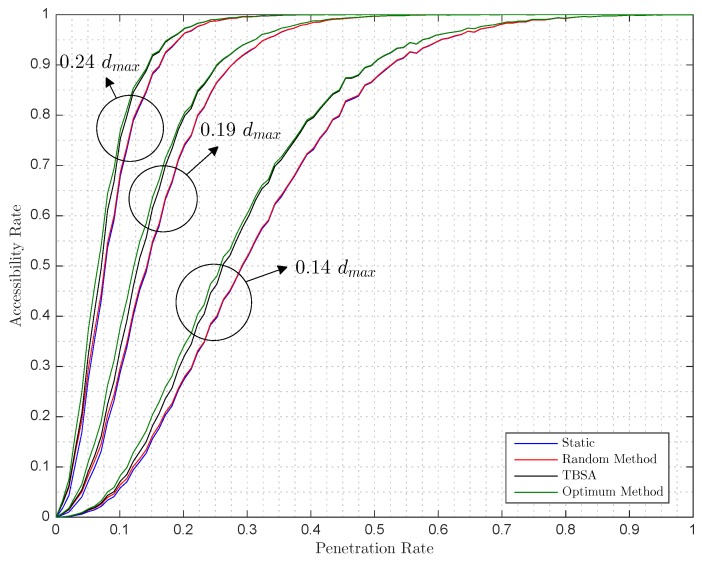
Simulated accessible rate versus penetration rate with an occupancy rate of 80% for a radius$/d_{max}$ of (*i*) 24%, (ii) 19% and (iii) 14%.

With a defined occupancy rate of 80%, Figure 6 and Figure 7 show how accessibility rate varies, on the one hand, while the ratio between the communication range and the maximum distance of the parking grows for a fixed penetration rate of 5%, 10% and 25%; on the other hand, the penetration rate varies for three fixed ratios, between the communication range and the maximum distance of the parking, of 14%, 19% and 24%. Given the static $A_{rate}$ by the current parking state, the random, optimum and TBSA are computed to further evaluate how $A_{rate}$ behaves. Note that, here, only a new autonomous car enters the parking lot, so the results shown are the improvements of the methods for the case of adding one autonomous car.

In Figure 6, when the ratio radius$/d_{max}$ is near 80%, the $A_{rate}$ equals 1, which means that the whole parking is accessible for the upcoming autonomous cars. However, this is only in this specific case for the simulated parameters of occupancy rate and penetration rate. For any fixed parameters of occupancy rate and penetration rate, there will be a ratio radius$/d_{max}$, where the $A_{rate}$ equals 1 determined by the size of the parking lot. Similarly, in Figure 7, the values of the occupancy rate and ratio radius$/d_{max}$ will determine the minimum value of the penetration rate that achieves $A_{rate}=1$.

It is interesting to remark that the accessibility rate can degenerate if the new autonomous car placed at a given parking spot does not make a new place accessible while occupying one that was previously free. This can easily be seen following the next example: if of the total 300 parking spaces, 10 are free and two of these are accessible by a new autonomous car, $A_{rate}$ is thus 2/10. Now, if a new autonomous car arrives and parks in a free and accessible parking space and does not make a new parking space accessible for another autonomous car, then there are nine free and one accessible parking spaces, so now $A_{rate}$ is 1/9, lower than before.

Comparing the aforementioned methods, it can be seen in Figure 6 and Figure 7 that the improvement of the random method over the static method is small for all of the cases. In contrast, our designed TBSA method approximates to the optimal method for all cases. Quantitatively, Table 1 summarizes that and presents the obtained $A_{rate}$ for different values of the occupancy rate, the penetration rate and the ratio radius$/d_{max}$. For example, for an occupancy rate of 80%, a penetration rate of 10% and a ratio radius$/d_{max}$ of 24% the $A_{rate}$ before the introduction of any new car is 67.98%. From the optimum method we know that the $A_{rate}$ can be improved up to 77.1%. The TBSA obtains an $A_{rate}$ of 75.45%, which means that it improved 81.91% of the maximum possible improvement, which is the improvement rate, whereas the random method only achieves 8.66% of the maximum improvement. Similarly, for $O_{rate}=0.8$, $P_{rate}=0.25$ and ratio radius$/d_{max}=0.19,$ the TBSA achieves 96.08% of the maximum improvement, whereas the random method only achieves 2.8%.

As expected, in Figure 6, as the ratio $radius/d_{max}$ increases, so does the accessibility rate due to anchors having larger coverage areas; thus, it is more likely that a free parking space and the road that leads to it will be covered by two or more areas of coverage of the anchors. In addition, these graphs present a staggered form due to the specific topology of the parking since the centers of the parking spaces are separated 2.5 m, and, therefore, increasing the radius a little does not guarantee covering a whole parking space, that is, to win a new accessible parking space and increase accessibility rate. On the other hand, in Figure 7, while the proportion of autonomous cars against traditional cars grows, the accessibility rate also grows due to the increased number of anchors covering a larger part of the parking area.

**Figure 8:**
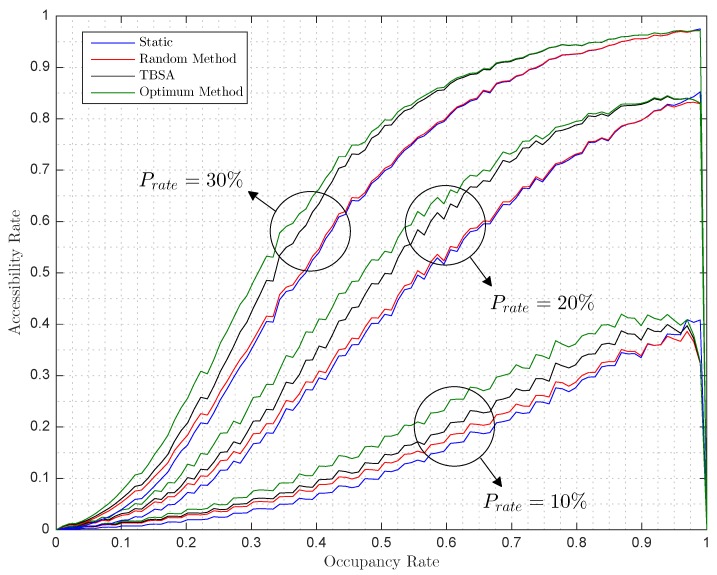
Simulated accessible rate versus occupancy rate with $radius/d_{max}=0.19$ for a penetration rate $P_{rate}$ of (*i*) 10%, (ii) 20% and (iii) 30%.

Figure 8 shows how the $A_{rate}$ grows as a function of the occupation of the parking lot. It is worth mentioning that, for occupancy rate values close to 100%, it is not possible to increase the accessibility rate because all free available spaces are covered by two or more anchors so these spaces are all accessible. In that situation, when a new autonomous car parks, the accessibility rate will decrease below the static accessibility rate and finally to zero, as it happens in Figure 8.

Notice that the designed tree based searching algorithm obtain increments in the accessibility rate similar to the optimum ones for all the parameters tested. Furthermore, this increment is achieved with a low complex algorithm—in contrast with the optimum method that computes the $A_{rate}$ for all the free accessible parking places.

**Figure 9:**
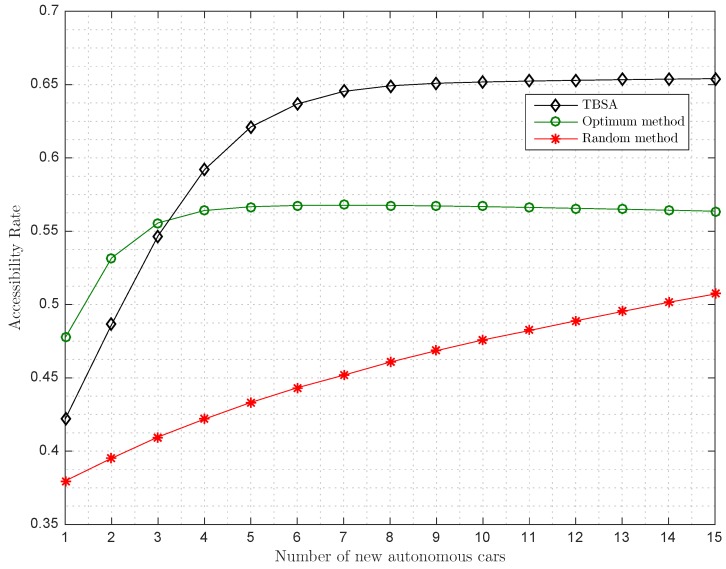
Simulated entrance of successive autonomous cars for $radius/d_{max}=0.24$, $O_{rate}=0.5$ and $P_{rate}=0.2$.

Since now, we have evaluated the performance of the methods in terms of $A_{rate}$ after the addition of one more autonomous car in the parking lot. In order to see the performance of the systems when more autonomous cars enter the parking lot, we modified the simulations to include *n* consecutive autonomous cars, that is, we generate a stationary parking. Then, we introduce one by one different autonomous cars in the places selected by the algorithms, and, finally, we evaluate the $A_{rate}$ as a function of the number of additional autonomous cars. The results of this new scenario are depicted in Figure 9. We can observe how the algorithms increase the $A_{rate}$ with the number of cars until they reach a stationary state where the $A_{rate}$ cannot be longer improved. Note that, if the autonomous car occupies a parking place and thus does not increase the number of accessible parking places, the $A_{rate}$ will decrease. If we compare the methods, we can observe that the TBSA outperforms the optimum method after the inclusion of four new cars. Note that the optimum method is computed as before and it is not the optimum method when taking into account the best placement of *n* successive autonomous cars. The computation of the optimum for *n* successive autonomous cars is an NP-hard problem as it involves the iterations over all the possible combinations of placing *n* successive cars into the available places, taking into account that the introduction of a new autonomous car will modify the available places. Again, the performance of the random method is far from the optimum method as expected.

From the simulated results, we evaluated the performance of the designed TBSA. We can conclude that the designed algorithm is close to the optimum method in the case of including a new autonomous car and it can perform better than the optimum method when successive autonomous cars enter the parking lot. This is due to the tree based structure employed that grants access to different branches of the parking, and in this way, improves the accessibility rate.

## 5. Conclusions

In this work, we have proposed a new parking scheme based on the cooperation of autonomous cars within a Vehicular Sensor Network (VSN). In particular, we designed a smart parking system on top of a VSN that allows the autonomous and traditional cars to coexist in the same parking lot. The best parking place for the autonomous car is selected by the system and the car is guided to the assigned place, thanks to a cooperative positioning approach that employs the stationary autonomous cars as anchor nodes. The best parking place is selected using the designed tree based searching algorithm (TBSA). The TBSA selects the best parking place according to a common good criterion that maximizes the accessibility for new autonomous cars to the parking lot. Furthermore, the optimum parking place is also computed and the behavior of our system is compared to the optimum case in a simulated environment that copies the structure of the parking lot of the Engineering School at *Universitat Autònoma de Barcelona*. The results show that the behavior of the TBSA is close to the optimum for the case of introducing one more autonomous car and outperforms the optimum method when successive autonomous cars enter the parking lot.

## Figures and Tables

**Table 1:** Simulated results in terms of $A_{rate}$ and improvement rate versus occupancy rate of the parking lot, penetration rate and communication range.

			Static	Random	TBSA	Optimum	Improvement Rate	
							Random	TBSA
$O_{rate}=0.3$	$radius/d_{max}=0.14$	$P_{rate}=0.05$	0.0015	0.0036	0.0038	0.0042	0.7778	0.8519
		$P_{rate}=0.10$	0.0058	0.0103	0.0110	0.0128	0.6429	0.7429
		$P_{rate}=0.25$	0.0453	0.0563	0.0644	0.0779	0.3374	0.5859
	$radius/d_{max}=0.19$	$P_{rate}=0.05$	0.0107	0.0177	0.0192	0.0233	0.5556	0.6746
		$P_{rate}=0.10$	0.0402	0.0543	0.0603	0.0762	0.3917	0.5583
		$P_{rate}=0.25$	0.2533	0.2697	0.3095	0.3646	0.1473	0.5049
	$radius/d_{max}=0.24$	$P_{rate}=0.05$	0.0431	0.0623	0.0650	0.0826	0.4861	0.5544
		$P_{rate}=0.10$	0.1733	0.2023	0.2159	0.2653	0.3152	0.4630
		$P_{rate}=0.25$	0.6248	0.6363	0.7060	0.7291	0.1103	0.7785
$O_{rate}=0.5$	$radius/d_{max}=0.14$	$P_{rate}=0.05$	0.0037	0.0067	0.0071	0.0079	0.7143	0.8095
		$P_{rate}=0.10$	0.0186	0.0257	0.0285	0.0339	0.4641	0.6471
		$P_{rate}=0.25$	0.1647	0.1744	0.2053	0.2341	0.1398	0.5850
	$radius/d_{max}=0.19$	$P_{rate}=0.05$	0.0247	0.0358	0.0390	0.0483	0.4703	0.6059
		$P_{rate}=0.10$	0.1154	0.1331	0.1496	0.1855	0.2525	0.4879
		$P_{rate}=0.25$	0.5603	0.5669	0.6522	0.6790	0.0556	0.7742
	$radius/d_{max}=0.24$	$P_{rate}=0.05$	0.1088	0.1361	0.1436	0.1805	0.3808	0.4854
		$P_{rate}=0.10$	0.3965	0.4160	0.4665	0.5184	0.1600	0.5742
		$P_{rate}=0.25$	0.8918	0.8942	0.9249	0.9271	0.0680	0.9377
$O_{rate}=0.8$	$radius/d_{max}=0.14$	$P_{rate}=0.05$	0.0110	0.0137	0.0147	0.0165	0.4909	0.6727
		$P_{rate}=0.10$	0.0588	0.0651	0.0718	0.0833	0.2571	0.5306
		$P_{rate}=0.25$	0.3976	0.4008	0.4642	0.4800	0.0388	0.8083
	$radius/d_{max}=0.19$	$P_{rate}=0.05$	0.0714	0.0831	0.0911	0.1102	0.3015	0.5077
		$P_{rate}=0.10$	0.2902	0.2999	0.3372	0.3802	0.1078	0.5222
		$P_{rate}=0.25$	0.8652	0.8662	0.8995	0.9009	0.0280	0.9608
	$radius/d_{max}=0.24$	$P_{rate}=0.05$	0.2739	0.2939	0.3222	0.3716	0.2047	0.4944
		$P_{rate}=0.10$	0.6798	0.6877	0.7545	0.7710	0.0866	0.8191
		$P_{rate}=0.25$	0.9875	0.9877	0.9904	0.9906	0.0645	0.9355
